# Ultra Performance Liquid Chromatography-Tandem Mass Spectrometry-Based Metabolomics Reveals Metabolic Alterations in the Mouse Cerebellum During *Toxoplasma gondii* Infection

**DOI:** 10.3389/fmicb.2020.01555

**Published:** 2020-07-10

**Authors:** Jun Ma, Jun-Jun He, Jun-Ling Hou, Chun-Xue Zhou, Hany M. Elsheikha, Xing-Quan Zhu

**Affiliations:** ^1^ State Key Laboratory of Veterinary Etiological Biology, Key Laboratory of Veterinary Parasitology of Gansu Province, Lanzhou Veterinary Research Institute, Chinese Academy of Agricultural Sciences, Lanzhou, China; ^2^ Department of Parasitology, School of Basic Medical Sciences, Shandong University, Jinan, China; ^3^ Faculty of Medicine and Health Sciences, School of Veterinary Medicine and Science, University of Nottingham, Loughborough, United Kingdom

**Keywords:** cerebellum, *Toxoplasma gondii*, metabolomics, pathway analysis, host-parasite interaction

## Abstract

*Toxoplasma gondii* is a protozoan parasite with a remarkable neurotropism. We recently showed that *T. gondii* infection can alter the global metabolism of the cerebral cortex of mice. However, the impact of *T. gondii* infection on the metabolism of the cerebellum remains unknown. Here we apply metabolomic profiling to discover metabolic changes associated with *T. gondii* infection of the mouse cerebellum using ultra performance liquid chromatography-tandem mass spectrometry (UPLC-MS/MS). Multivariate statistics revealed differences in the metabolic profiles between the infected and control mouse groups and between the infected mouse groups as infection advanced. We also detected 10, 22, and 42 significantly altered metabolites (SAMs) in the infected cerebellum at 7, 14, and 21 days post infection (dpi), respectively. Four metabolites [tabersonine, arachidonic acid (AA), docosahexaenoic acid, and oleic acid] were identified as potential biomarker or responsive metabolites to *T. gondii* infection in the mouse cerebellum. Three of these metabolites (AA, docosahexaenoic acid, and oleic acid) play roles in the regulation of host behavior and immune response. Pathway analysis showed that *T. gondii* infection of the cerebellum involves reprogramming of amino acid and lipid metabolism. These results showcase temporal metabolomic changes during cerebellar infection by *T. gondii* in mice. The study provides new insight into the neuropathogenesis of *T. gondii* infection and reveals new metabolites and pathways that mediate the interplay between *T. gondii* and the mouse cerebellum.

## Introduction

*Toxoplasma gondii* is an apicomplexan protozoan pathogen that can infect nearly all warm-blooded vertebrate animals and humans. In general, infection by *T. gondii* can be asymptomatic or causes mild non-specific symptoms in immunocompetent people. However, in immunocompromised patients, the consequences of *T. gondii* infection can be fatal. One-third of the world population has been estimated to be chronically infected by *T. gondii*, and the global seropositive rates range from 0% to over 90%, with the highest seropositive rates reported in Latin America, South America, and the Middle East (Pappas et al., 2009). Infection by *T. gondii* has been reported to cause behavioral changes in rodents (Ingram et al., 2013; Evans et al., 2014; Tyebji et al., 2019) and humans (Ustun et al., 2004; Shapira et al., 2012; Elsheikha and Zhu, 2016). A recent study showed that neuroinflammation induced by *T. gondii* may underlie the behavioral alterations in mice (Boillat et al., 2020). Also, *T. gondii* infection impairs the GLT-1-dependent glutamate transportation and redistributes glutamate decarboxylase to the postsynaptic neuron cytosol, resulting in excitotoxicity of postsynaptic neurons (David et al., 2016; Mendez and Koshy, 2017).

Cerebellum is an essential part of the brain, which controls mood, feeling, learning, thinking, motor coordination, temporal discrimination, and food-anticipatory activity (Mendoza et al., 2010). Cerebellar damage can impair these functions and results in ataxia, dyslexia, vertigo, and learning disorders (Reeber et al., 2013; Abdoli and Dalimi, 2014). Signaling pathways mediated by neurotransmitters, such as GABAergic and glutamatergic pathways, are crucial for the cerebellum functions (De Zeeuw et al., 2011). During *T. gondii* infection, alterations of some neurotransmitters, such as dopamine, tryptophan, which is a precursor of serotonin, kynurenine, and quinolinic acid, have been shown to contribute to the changes in the host behavior (Elsheikha et al., 2016). *T. gondii* has been also shown to impact the metabolism of the host cell *via* usurping and modulating host metabolites to potentiate parasite replication (Zhou et al., 2015, 2016, 2017, 2019; Chen et al., 2017, 2018; Ma et al., 2019). In a previous metabolomics study, we showed that the levels of neurotransmitter in the mouse cerebral cortex are altered by *T. gondii* infection (Ma et al., 2019). Since neurologic defects detected in *T. gondii*-infected animals could be also attributed to the brain cerebellum dysfunction, knowledge of the cerebellum metabolomic changes during *T. gondii* infection may improve the understanding of the mechanisms that underpin the neurobehavioral alterations attributed to *T. gondii*.

In this study, ultra performance liquid chromatography-tandem mass spectrometry (UPLC-MS/MS) based metabolomics analysis was used to detect the metabolic changes that occur in the mouse cerebellum after *T. gondii* infection. This approach enabled the identification of significantly altered metabolites and associated pathways in the cerebellar tissue of infected compared to non-infected mice at 7, 14, and 21 days post infection (dpi).

## Materials and Methods

### Mice and *Toxoplasma gondii* Infection

Three-week-old female BALB/c mice (*n* = 36) were purchased from Lanzhou University Laboratory Animal Center (Lanzhou, China). Mice were separated into six groups (six mice/group). The mice in the infected groups were orally gavaged with 10 *T. gondii* cysts of Pru strain suspended in 0.5 ml phosphate-buffered saline (PBS). Mice in the control groups were sham-treated with 0.5 ml PBS only without parasite cysts. All mice were provided non-medicated feed and water *ad libitum* during the experiment. The mice were monitored twice daily for signs of illness and mortality. At 7, 14, and 21 dpi, mice from infected and control groups were sacrificed by CO_2_ asphyxiation, and the cerebellum of each mouse was immediately dissected out with scissors and forceps.

### Cerebellum Collection and Confirmation of Infection

The mouse cerebella were identified according to anatomical atlas of mice brains. The cerebella were collected from infected and control (non-infected) mice (six mice/groups) at 7, 14, and 21 dpi. The collected cerebella were washed with chilled PBS three times to remove contaminating blood and stored at −80°C until used for metabolite or DNA extraction. Approximately 10 mg of each collected cerebellum was used for DNA extraction. DNA of each sample was extracted using TIANamp Genomic DNA kit (TianGen™, Beijing, China) according to the manufacturer’s instructions. The presence of *T. gondii* in the cerebellum was tested using PCR, and primers that target *B1* gene of *T. gondii*: B1F: 5'-TGCATAGGTTGCAGTCACTG-3', and B1R: 5'-TCTTTAAAGCGTTCGTGGTC-3'. The PCR amplification was performed as follows: an initial denaturation at 95°C for 5 min followed by 35 cycles at 95°C for 10 s, 60°C for 10 s, and 72°C for 20 s. Negative control sample (PBS only) and positive control samples (*T. gondii* DNA) were included in each PCR run. PCR amplification products were analyzed by 2% agarose gels, and PCR bands were observed under a UV illuminator.

### Extraction of Metabolites

Before the experiment, the cerebellar tissues stored at −80°C were thawed gradually by incubation at −20°C for 30 min, followed by incubation on ice at 4°C. Approximately 25 mg of each defrosted cerebellum was used for metabolite extraction. Each defrosted cerebellum was mixed with 800 μl H_2_O/50% MeOH (vol/vol), and then lysed with TissueLyser bead-mill homogenizer (Qiagen, Hilden, Germany). The cerebellum homogenate was centrifuged at 25,000 *g* for 20 min at 4°C. The cerebellum homogenate supernatant was transferred into new tubes, and 50 μl of the supernatants was loaded into solid phase extraction (SPE) column for extracting the metabolites. The extracted metabolites were dissolved in acetonitrile. A quality control (QC) sample was made by mixing equal volumes (20 μl) from each processed cerebellar sample and used to represent all the metabolites encountered during analysis to assess the reproducibility and reliability of the UPLC-MS/MS method. Metabolites extracted from all cerebellar samples were stored at −80°C until use.

### LC-MS/MS Analysis for Untargeted Metabolite Profiling

Ultra performance liquid chromatography (UPLC) system (Waters, UK) was used for unbiased (global) metabolomics analysis of all cerebellar samples. An ACQUITY UPLC BEH C18 column (100 × 2.1 mm, 1.7 μm, Waters, UK) was used for the reversed phase separation of metabolites. The column oven was maintained at 50°C. The flow rate was 0.4 ml/min, and the mobile phase consisted of solvent A (water + 0.1% formic acid) and solvent B (acetonitrile + 0.1% formic acid). The following gradient used for metabolite elution was applied: 100% solvent A for 0–2 min; 0–100% solvent B for ~11 min; 100% solvent B for 11–13 min; and 100% solvent A for 13–15 min. The eluted metabolites were further analyzed using high-resolution tandem mass spectrometer SYNAPT G2-XS QTOF (Waters, Ireland) in the negative electrospray ionization (ESI−) and positive electrospray ionization (ESI+) modes. The TOF mass range was set from 50 to 1,200 Da, and the scan time was 0.2 s. For the MS/MS detection, all precursors were fragmented using 20–40 eV, and the scan time was set to 0.2 s. For calibrating the mass accuracy, during the acquisition, the LE signal was acquired every 3 s. Centroid mean square error (MSE) mode was used for collection of the mass spectrometry data.

### Metabolite Identification, Pathway Enrichment, and Multivariate Statistical Analysis

Progenesis QI software was used for identification of the cerebellum metabolites. The mass-to-charge ratio (m/z) and retention time of the metabolites were used for metabolite identification. For validation and confirmation, the metabolites, MS/MS spectra, molecular mass data, and retention times of metabolites were compared against standard substances. Student’s *t*-test was used for the identification of significantly altered metabolites (SAMs) based on values of *p* < 0.05. The log_2_ fold change (log_2_FC) represented the ratio between abundance of the average ion intensities in the infected cerebella compared to the non-infected cerebella.

The identified SAMs were annotated using human metabolome database (HMDB^1^) and Kyoto encyclopedia of genes and genome (KEGG^2^) to determine the enriched metabolic pathways. Cerebellum metabolite abundances were used as input data for partial least squares-discriminant analysis (PLS-DA) to discriminate infected cerebellar samples from control samples, and PLS-DA was performed using SIMCA 13.0 software. A heat-map was used to show the relatively disturbed and unbalanced metabolic state among infected cerebellar samples compared to samples of control mice. Receiver operating characteristic (ROC) analysis was performed to identify potential biomarker or responsive metabolite to *T. gondii* infection. ROC curve and the area under the curve (AUC) of ROC were analyzed using the pROC R package (Robin et al., 2011).

## Results

### *Toxoplasma gondii* Infection in Mice Cerebella

**Figure 1:**
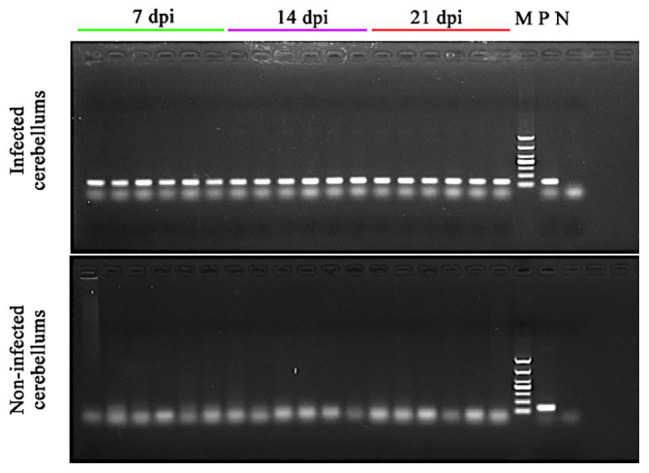
Confirmation of the presence of *Toxoplasma gondii* infection in the cerebella of the infected mice. DNA was extracted from the cerebellum of infected and non-infected mice at 7, 14, and 21 dpi and used in PCR to detect *T. gondii B1* gene. 7 dpi (lanes 1–6), 14 dpi (lanes 7–12), and 21 dpi (lanes 13–18): PCR amplicons from the cerebellum of infected (top image) and non-infected (bottom image) mice; M, DNA marker; P, *T. gondii* PCR positive control; and N, PCR negative control.

At 7 dpi, no significant clinical signs of toxoplasmosis were observed in all mice. At 14 dpi, mice in infected groups showed significant clinical signs, such as loss of appetite and ruffled fur, whereas the mice in control groups remained apparently healthy. At 21 dpi, infected mice seemed to regain their normal physical status, probably correlated with the development of the chronic infection stage. All cerebella of infected mice collected at 7, 14, and 21 dpi were *T. gondii B1* gene positive. However, no *B1* gene amplification product was detected in the cerebella of non-infected mice in the control groups (Figure 1).

### Metabolic Profiles of the Cerebella

**Figure 2:**
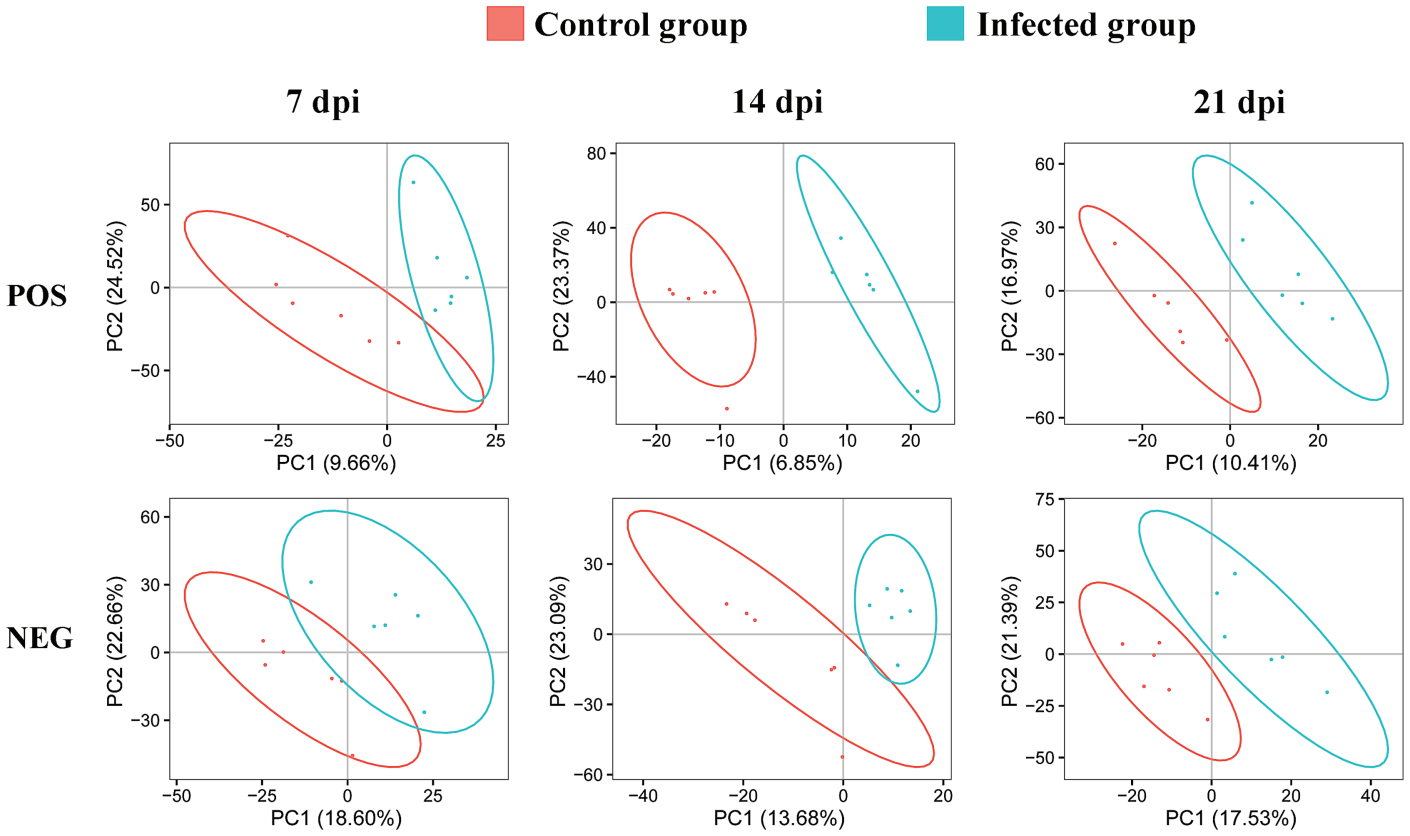
Two-dimensional partial least squares-discriminant analysis (PLS-DA) score plots of liquid chromatography-mass spectrometry metabolomic data showing the separation between infected and control mice at 7, 14, and 21 dpi in the positive (POS) and negative (NEG) ion modes. The ellipses enclose the 95% confidence intervals estimated by the sample means and covariances of each group.

We detected 3,200 and 6,198 metabolic ions in the ESI− mode and ESI+ mode, respectively. To reveal whether the metabolite profile of infected cerebellum was different from non-infected cerebellum, PLS-DA analysis was performed. As shown in Figure 2, the infected cerebellum samples and non-infected control cerebellum samples were clearly separated in the PLS-DA plot, and the separation between infected and non-infected cerebellum samples was more obvious at 14 and 21 dpi.

**Figure 3:**
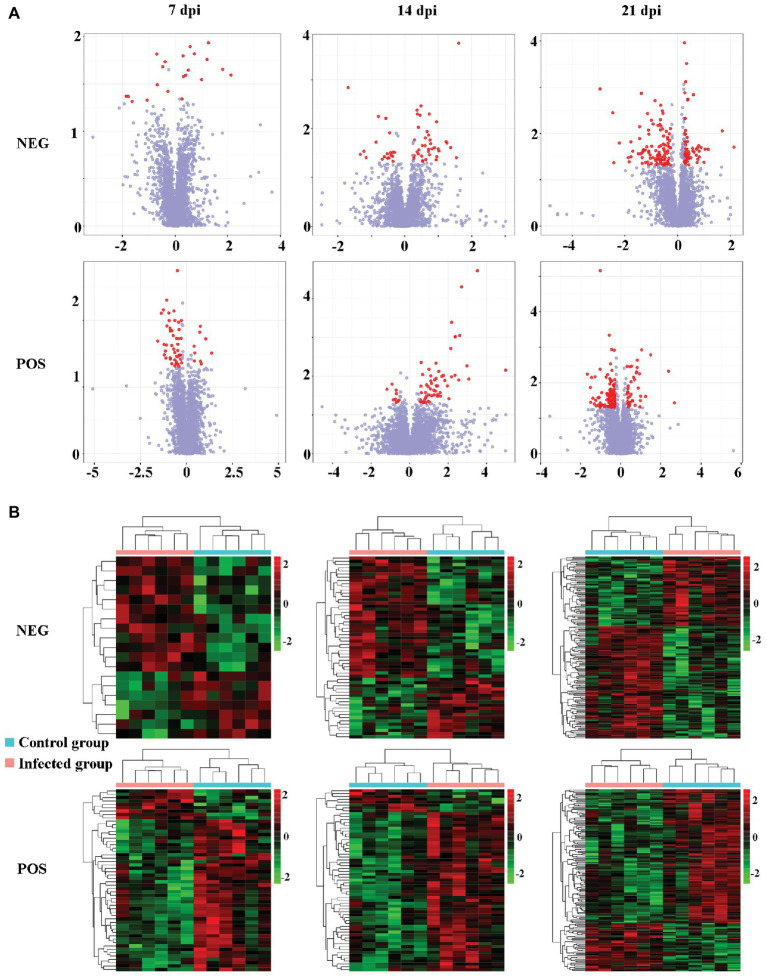
The intensity patterns and hierarchical cluster analysis of the differentially abundant metabolites between infected and non-infected mice in the positive and negative ion modes. **(A)** Volcano plots showing significantly differentially abundant ions denoted as red dots. The *y*-axis shows statistical significance values −log_10_(*p*) for the abundance of metabolite ions, and the *x*-axis shows the magnitude of the log_2_ fold change (log_2_FC) of metabolite ions between infected and non-infected samples. **(B)** Heat-map plots of the intensity of the differentially abundant metabolite ions showing significantly different metabolic profiles between infected and non-infected (control) cerebellum samples. Each row represents data for a specific metabolite, and each column represents a mouse (*T. gondii*-infected or healthy control). Different colors correspond to the different intensity levels of metabolites. Red and green colors represent increased and decreased levels of metabolites, respectively.

**Figure 4:**
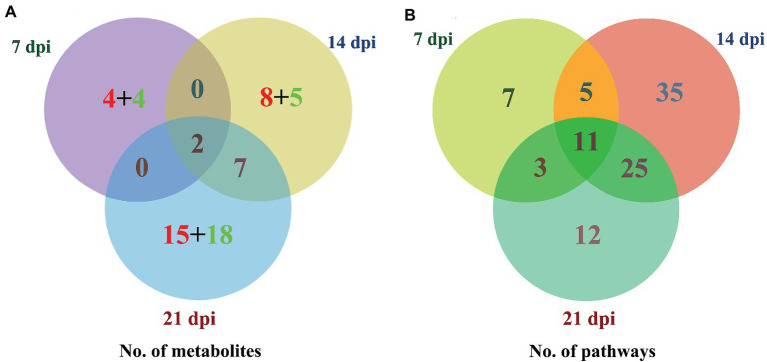
Three-way Venn diagrams of the **(A)** differentially abundant metabolites and **(B)** enriched metabolic pathways at 7, 14, and 21 dpi. The time after infection is indicated next to the corresponding circle. The numbers of metabolites shared between the groups are indicated at the intersections of the circles in the Venn diagram. The number of metabolites specific to each time point is shown inside the corresponding circle in red + green colors, denoting the upregulated and downregulated metabolites, respectively. Two SAMs and 11 enriched metabolic pathways were shared in common between all groups at the three time points after infection.

The levels of dozens of retention time-exact mass pairs were significantly affected in the cerebellum following *T. gondii* infection. In the ESI− mode, 21, 57, and 148 retention time-exact mass pairs were altered at 7, 14, and 21 dpi, respectively. However, in the ESI+ mode, the levels of 54, 63, and 154 retention time-exact mass pairs were significantly altered at 7, 14, and 21 dpi, respectively. The volcano and heat-map plots of these retention time-exact mass pairs are shown in Figure 3. Around 10, 22, and 42 SAMs were identified in infected cerebella at 7, 14, and 21 dpi, respectively. The details of SAMs at each time point are listed in Supplementary Table S1. Venn diagram showed that two SAMs (2-lysophosphatidylcholine and lecithin) were common in the infected cerebella at all time points after infection (Figure 4A). 2-Lysophosphatidylcholine and lecithin were downregulated at 7 and 21 dpi, but were upregulated at 14 dpi (Supplementary Table S1). Around 8, 13, and 33 metabolites were exclusively altered at 7, 14, and 21 dpi, respectively.

Four upregulated metabolites (pyroglutamic acid, 7(1)-hydroxychlorophyll a, 3,4-dihydroxyphenylacetic acid, and p-Cresol) and four downregulated metabolites (ceramide, cephalin, nerolidol, and oleic acid) were detected at 7 dpi only. Eight upregulated metabolites [diacylglycerol (DAG), galactosylsphingosine, arachidonic acid (AA), chitin, 17alpha, 21-dihydroxypregnenolone, sulfatide, 4,4-dimethyl-5a-cholesta-8-en-3b-ol, and Norfloxacin] and five downregulated metabolites (uridine, tabersonine, sphingomyelin, ethylbenzene, and 5alpha, cholesta-7,24-dien-3beta-ol) were exclusively detected at 14 dpi. There were 15 upregulated metabolites (chenodeoxycholic acid, traumatic acid, 2-chloro-3-oxoadipate, 9-OxoODE, phenethyl alcohol, S-lactoylglutathione, androstan-3alpha, 17beta-diol, arachidonate, 5-hydroxyconiferaldehyde, calcitetrol, allotetrahydrodeoxycorticosterone, 13-OxoODE, eucalyptol, cytidine, and phosphatidylethanolamine) and 18 downregulated metabolites [5,6-epoxytetraene, cortolone, pravastatin, 5,6-epoxy-8,11,14-eicosatrienoic acid, fexofenadine, rhodovibrin, palmitoleic acid, estriol, phosphatidic acid (PA), xanthoxin, sphingosine, 2-arachidonylglycerol, capric acid, 2'-*N*-acetylparomamine, tryptophol, (−) alpha-terpineol, vitamin A, and 1,2-dehydroreticuline] were exclusively detected at 21 dpi.

### Metabolic Pathways Affected by *Toxoplasma gondii*

**Figure 5:**
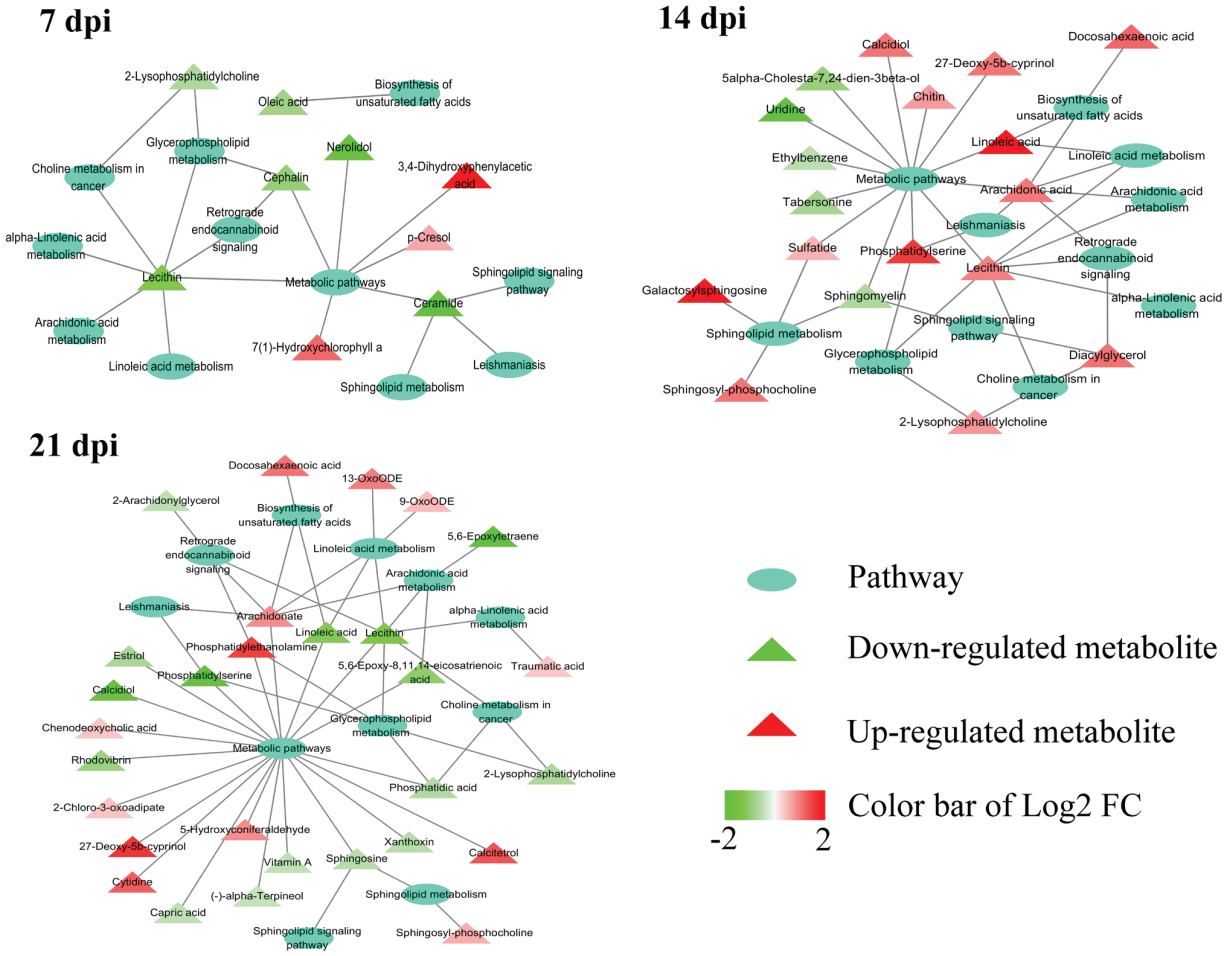
Relationship among 11 common pathways and SAMs. Oval denotes the common pathways among 7, 14, and 21 dpi. Triangle denotes the differentially abundant metabolites. Green and red color represents downregulated metabolites and upregulated metabolites, respectively. In the 11 pathways, 9, 18, and 29 metabolites were significantly altered at 7, 14, and 21 dpi, respectively.

As shown in Figure 4B, the SAMs were enriched in 26, 76, and 51 pathways at 7, 14, and 21 dpi, respectively. Details of enriched pathways are showed in Supplementary Table S1. Also, we found that 11 pathways were consistently affected throughout the study (i.e., at 7, 14, and 21 dpi). These shared pathways included sphingolipid metabolism, metabolic pathways, sphingolipid signaling pathway, leishmaniasis, glycerophospholipid metabolism, choline metabolism in cancer, AA metabolism, linoleic acid metabolism, alpha-linolenic acid metabolism, retrograde endocannabinoid signaling, and biosynthesis of unsaturated fatty acids (Table 1 and Figure 5). However, 7, 35, and 12 pathways were exclusively altered at 7, 14, and 21 dpi, respectively.

**Table 1:** The summary of significantly altered metabolite (SAM) of the 11 common pathways at 7, 14, and 21 days post infection (dpi).

Pathways	Number of significantly altered metabolites					
	7 dpi		14 dpi		21 dpi	
	Downregulated metabolite	Upregulated metabolite	Downregulated metabolite	Upregulated metabolite	Downregulated metabolite	Upregulated metabolite
Alpha-linolenic acid metabolism	1	0	0	1	1	1
Arachidonic acid (AA) metabolism	1	0	0	2	3	1
Biosynthesis of unsaturated fatty acids	1	0	0	3	1	2
Choline metabolism in cancer	2	0	0	3	3	0
Glycerophospholipid metabolism	3	0	0	3	4	1
Leishmaniasis	1	0	0	2	1	1
Linoleic acid metabolism	1	0	0	3	2	3
Metabolic pathways	4	3	5	8	13	8
Retrograde endocannabinoid signaling	2	0	0	3	2	2
Sphingolipid metabolism	1	0	1	3	1	1
Sphingolipid signaling pathway	1	0	1	1	1	0

At 7 dpi, all the seven pathways had one upregulated metabolite, including 7(1)-hydroxychlorophyll A (log_2_FC 0.756, *p* = 0.012) of porphyrin and chlorophyll metabolism, p-Cresol (log_2_FC 0.422, *p* = 0.024) of protein digestion and absorption, and 3,4-dihydroxyphenylacetic acid (DOPAC; log_2_FC 2.128, *p* = 0.025) of five pathways (tyrosine metabolism, dopaminergic synapse, cocaine addiction, amphetamine addiction, and alcoholism pathway). At 14 dpi, all the 35 pathways had one upregulated metabolite, such as chitin (log_2_FC 0.483, *p* = 0.003) of amino sugar and nucleotide sugar metabolism, norfloxacin (log_2_FC 0.334, *p* = 0.009) of ABC transporters, calcidiol (log_2_FC 0.691, *p* = 0.018) of tuberculosis, and DAG (log_2_FC 0.705, *p* = 0.027) of 32 pathways (EGFR tyrosine kinase inhibitor resistance, MAPK signaling pathway, ErbB signaling pathway, Ras signaling pathway, Rap1 signaling pathway, calcium signaling pathway, chemokine signaling pathway, NF-kappa B signaling pathway, HIF-1 signaling pathway, adrenergic signaling in cardiomyocytes, VEGF signaling pathway, gap junction, natural killer cell mediated cytotoxicity, T cell receptor signaling pathway, B cell receptor signaling pathway, circadian entrainment, long-term potentiation, glutamatergic synapse, cholinergic synapse, insulin secretion, estrogen signaling pathway, melanogenesis, thyroid hormone synthesis, thyroid hormone signaling pathway, endocrine and other factor-regulated calcium reabsorption, salivary secretion, gastric acid secretion, pancreatic secretion, carbohydrate digestion and absorption, African trypanosomiasis, glioma, and non-small cell lung cancer).

Twelve cerebellum pathways were exclusively altered at 21 days post *T. gondii* infection, including phenylalanine metabolism (Phenethyl alcohol—log_2_FC 0.391, *p* = 0.038), pyruvate metabolism (S-lactoylglutathione—log_2_FC 0.446, *p* = 0.043), glycerolipid metabolism (S-lactoylglutathione—log_2_FC 0.446, *p* = 0.043), bile secretion (chenodeoxycholic acid with log_2_FC 0.265, *p* = 0.041; pravastatin—log_2_FC –0.693, *p* = 0.020; fexofenadine—log_2_FC –0.596, *p* = 0.024), butirosin and neomycin biosynthesis (2'-*N*-acetylparomamine—log_2_FC –0.315, *p* = 0.020), phosphatidylinositol signaling system (PA—log_2_FC –0.403, *p* = 0.030), pancreatic cancer (PA—log_2_FC –0.403, *p* = 0.030), neuroactive ligand-receptor interaction (2-arachidonylglycerol—log_2_FC –0.337, *p* = 0.028), tryptophan metabolism (tryptophol—log_2_FC –0.297, *p* = 0.008), retinol metabolism (vitamin A—log_2_FC –0.312, *p* = 0.033), vitamin digestion and absorption (vitamin A—log_2_FC –0.312, *p* = 0.033), and apoptosis (sphingosine—log_2_FC –0.359, *p* = 0.028).

### Identification of Responsive Metabolites in the Infected Cerebellum

**Figure 6:**
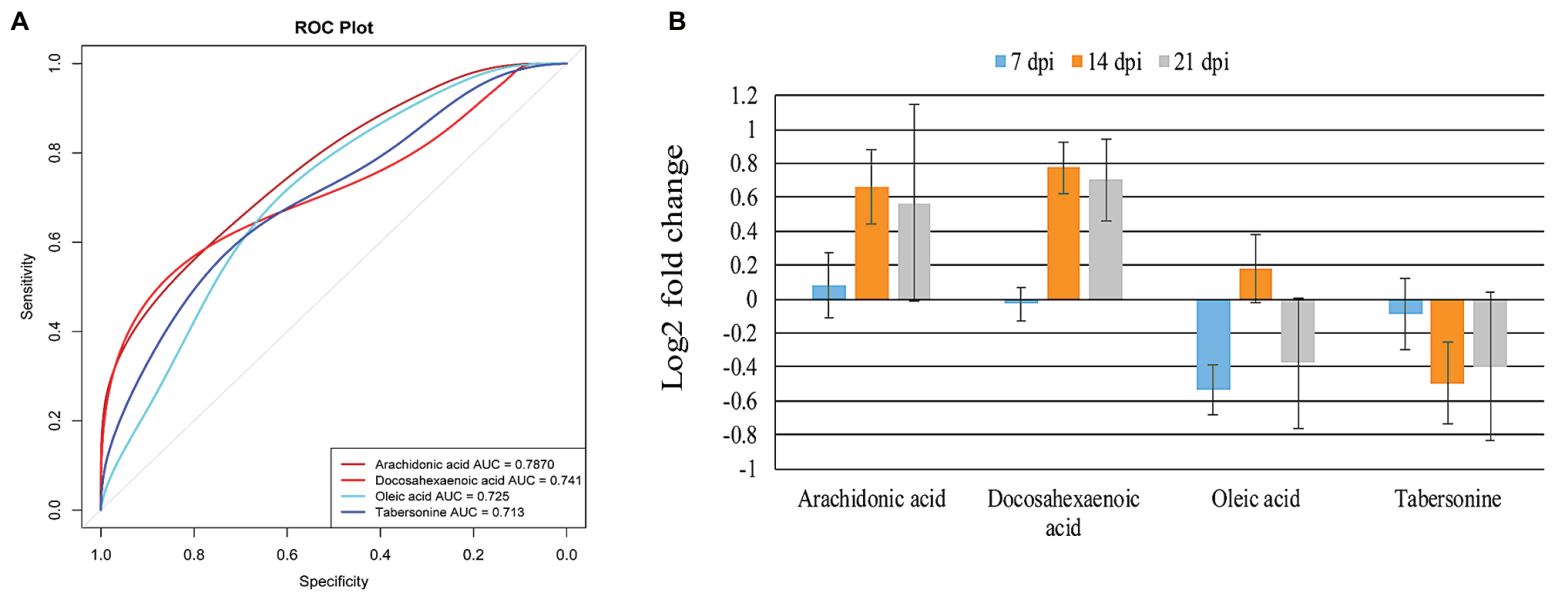
Identification and intensity of the potential biomarkers. **(A)** Receiver operating characteristic (ROC) analysis of four identified potential biomarkers or responsive metabolites in the infected mouse cerebellum. The four indicated metabolites show area under the curve (AUC) > 0.7. **(B)** Relative abundance of the four potential biomarkers or responsive metabolites in the infected cerebellum at 7, 14, and 21 dpi.

To identify *T. gondii* responsive metabolites in infected cerebella, ROC analysis was performed. As shown in Figure 6A, four metabolites (tabersonine, AA, docosahexaenoic acid, and oleic acid) showed good predictability of *T. gondii* infection in the mouse cerebellum with AUC > 0.7. Both AA and docosahexaenoic acid were upregulated in the mouse cerebellum; however, oleic acid and tabersonine were downregulated (Figure 6B).

### Discussion

In this study, we used UPLC-MS/MS based metabolomics to understand how the metabolic profiles of the mouse cerebellum change during *T. gondii* infection. Our PLS-DA results showed that the metabolic profiles between infected and non-infected cerebella were different (Figure 2), and the separation became more obvious as infection advanced. We also investigated whether different time points after infection have unique metabolomic signatures, which may inform on infection progression. Our analysis identified 8, 13, and 33 SAMs exclusively at 7, 14, and 21 dpi, respectively. Two metabolites (2-lysophosphatidylcholine and lecithin) were altered at all three time points. 2-Lysophosphatidylcholine plays a role in phospholipid metabolism and in maintaining the integrity of the cell membrane (van den Bosch, 1974). A previous report showed that the production of 2-lysophosphatidylcholine, which activates T lymphocytes, is triggered by antigen stimulation, and that 2-lysophosphatidylcholine production is time dependent (Asaoka et al., 1992). Lecithin has been used as an adjuvant to enhance cellular immune response to antigen stimulation (Kawano and Noma, 1995; Sloat et al., 2010; Gasper et al., 2016). These two metabolites were downregulated at 7 and 21 dpi; however, they were upregulated at 14 dpi. Interestingly, both metabolites are involved in glycerophospholipid metabolism pathway, which plays a role in neurological disorders (Farooqui et al., 2000). Whether alterations of these two metabolites contribute to the changes of host behavior during *T. gondii* infection remain to be determined.

As shown in Figure 4B, 11 pathways were altered at 7, 14, and 21 dpi, whereas 7, 35, and 12 pathways were exclusively altered at 7, 14, and 21 dpi, respectively. The DOPAC is an oxidation product of the neurotransmitter dopamine, which is a critical molecule for regulating learning and motivation (Berke, 2018). Several studies showed that schizophrenia is linked with upregulation of dopamine signaling (Frankle and Laruelle, 2002; Nikolaus et al., 2009; Beaulieu and Gainetdinov, 2011) and *T. gondii* infection (Elsheikha and Zhu, 2016). In the present study, DOPAC was significantly upregulated at 7 dpi only (log_2_FC 2.128, *p* = 0.025) which agrees with the result of a previous study (Prandovszky et al., 2011). Upregulation of dopamine metabolic process results in downregulation of dopamine. In the present study, the dopamine was slightly upregulated (log_2_FC 0.091, *p* = 0.293). Although the mechanism underlying this result remains unknown, *T. gondii* encodes a tyrosine hydroxylase, which participates in the synthesis of dopamine. The dopamine production derived from *T. gondii* could be a source to replenish the cerebellar dopamine (McConkey et al., 2013, 2015) resulting in slightly upregulated dopamine in the infected mouse cerebellum. The relationships among alteration of dopamine pathway, *T. gondii* and schizophrenia need to be elucidated. Pathway analysis showed that this metabolite is involved in five neural-related metabolic pathways, including tyrosine metabolism, dopaminergic synapse, cocaine addiction, amphetamine addiction, and alcoholism pathway, suggesting that significant neuro-metabolic alterations may occur in the mouse cerebellum during acute *T. gondii* infection. This result agrees with a previous metabolomic profiling of mouse sera (Zhou et al., 2017).

At 14 dpi, immune-related pathways became more prominent, such as MAPK signaling pathway, chemokine signaling pathway, natural killer cell mediated cytotoxicity, NF-kappa B signaling pathway, T cell receptor signaling pathway, and B cell receptor signaling pathway. Interestingly, 35 pathways were exclusively altered at 14 dpi, and all the pathways had one upregulated DAG (log_2_FC 0.705, *p* = 0.027). DAG is a component of the cell membrane and a key lipid secondary messenger for immune system. The production and clearance of DAG is coordinated by the host to offset pathogen infection (Carrasco and Merida, 2007; Shahnazari et al., 2010). The deletion of DAG kinase zeta that catalyzes the conversion of DAG to PA impaired Th1 immune responses and increased susceptibility to *T. gondii* infection (Liu et al., 2007), suggesting that DAG plays a role in the host immune defense against *T. gondii*. Thus, upregulation of DAG may promote the immune response against *T. gondii* in mouse cerebellum at 14 dpi. This result provides a new clue for understanding how *T. gondii* infection is controlled in the mouse cerebellum.

Compared with 7 and 14 dpi, 12 pathways were exclusively altered at 21 dpi, five of which participate in various metabolic pathways, including phenylalanine metabolism, pyruvate metabolism, glycerolipid metabolism, retinol metabolism, and tryptophan metabolism. Alterations in the metabolism of phenylalanine, retinol, and tryptophan can lead to behavioral changes in the host (Elsheikha et al., 2016; Elsheikha and Zhu, 2016). Tryptophan is a precursor of neurotransmitters (serotonin and melatonin) and neuroactive substances (Young, 1996). Dysregulation of tryptophan is associated with neuropsychiatric disorders, such as epilepsy, multiple sclerosis, and schizophrenia (Ravikumar et al., 2000). Low phenylalanine impaired mouse behavior and reduced the levels of brain neurotransmitter (Sawin et al., 2014). Retinol, also known as vitamin A, participates in the regulation of mouse mood and behavior (O’Reilly et al., 2008; Buxbaum et al., 2014). Tryptophol and phenethyl alcohol are the metabolic products of tryptophan and phenylalanine, respectively. At 21 dpi, tryptophol (log_2_FC –0.297, *p* = 0.008) and vitamin A (log_2_FC –0.312, *p* = 0.033) were downregulated in the infected mouse cerebellum, whereas phenethyl alcohol (log_2_FC 0.391, *p* = 0.038) was upregulated. Additionally, there was another downregulated metabolite, 2-arachidonylglycerol (log_2_FC –0.337, *p* = 0.028), which is one of the endogenous cannabinoid-receptor agonists that regulate many neurobehavioral functions (Iannotti et al., 2016).

At the three time points post infection, 11 pathways were altered in all examined mouse cerebellum, including alpha-linolenic acid metabolism, AA metabolism, biosynthesis of unsaturated fatty acids, choline metabolism in cancer, glycerophospholipid metabolism, leishmaniasis, linoleic acid metabolism, metabolic pathways, retrograde endocannabinoid signaling, sphingolipid metabolism, and sphingolipid signaling pathway. Interestingly, most of these pathways had downregulated metabolites at 7 dpi and upregulated metabolites at 14 dpi (Table 1 and Figure 5). AA metabolism, choline metabolism in cancer and glycerophospholipid metabolism were downregulated at 21 dpi (Table 1 and Figure 5). These three pathways were also found to be downregulated in mouse cerebral cortices infected by *T. gondii* (Ma et al., 2019). The metabolites of AA metabolism pathway were upregulated at 14 dpi, but were downregulated at 7 and 21 dpi. AA is an n-6 polyunsaturated fatty acid that activates host inflammatory response (Grimble and Tappia, 1998; Levick et al., 2007). In addition to immune regulation, AA and its metabolites participate in regulating some neural functions (Shimizu and Wolfe, 1990). For example, AA can activate protein kinase C (PKC; Shearman et al., 1989) that regulates neurogenesis (Cambray-Deakin et al., 1990) and neurite outgrowth (Herrick-Davis et al., 1991).

The AA has been identified as a potential biomarker in mouse spleen (Chen et al., 2017), liver (Chen et al., 2018), and cerebral cortex (Ma et al., 2019), following infection by *T. gondii*. In the present study, AA was also identified as potential biomarker of cerebellum infection by *T. gondii* (Figure 6A). Using ROC analysis, four metabolites (AA, docosahexaenoic acid, oleic acid, and tabersonine) had AUC value > 0.7, suggesting that these four metabolites can be valuable biomarkers or responsive metabolites to *T. gondii* infection of the cerebellum. AA and docosahexaenoic acid were upregulated in the infected cerebellum (Figure 6B). AA, tabersonine, docosahexaenoic acid, and oleic acid play immunoregulatory roles in mice. As mentioned above, AA activates host inflammatory response (Grimble and Tappia, 1998; Levick et al., 2007). Tabersonine can protect the lung from acute injury *via* inhibiting ubiquitination of the tumor necrosis factor receptor (TNFR)-associated factor 6 (TRAF6), which plays immuno-inflammatory roles (Zhang et al., 2018). Thus, downregulation of tabersonine may enhance the immune response to clear *T. gondii* in cerebellum. Docosahexaenoic acid (n-3 polyunsaturated fatty acid) and oleic acid (monounsaturated fatty acid) can decrease the responsiveness to cytokines (Grimble and Tappia, 1998). Taken together, these data show that AA, docosahexaenoic acid, oleic acid, and tabersonine are promising candidates for further elucidation of the interaction between host and *T. gondii* in the mouse cerebellum.

## Conclusions

In this study, we successfully applied global metabolomics to investigate the differences in the metabolic profiles between *T. gondii*-infected mice and non-infected mice. Two immunoregulatory metabolites (2-lysophosphatidylcholine and lecithin) were significantly altered during the entire course of *T. gondii* infection. We identified differential metabolites related to the metabolism of lipids (e.g., glycerophospholipid) and amino acid (phenylalanine, retinol, and tryptophan), which play roles in neuropsychiatric disorders. Pathway enrichment analysis identified 11 pathways, mainly involved in lipid metabolism, which were altered in the infected mouse cerebellum at all time points. Four metabolites, including AA, tabersonine, docosahexaenoic acid, and oleic acid, were identified as potential infection responsive metabolites, and may have important implications in the diagnosis of cerebral toxoplasmosis. These four metabolites have immunoregulatory roles in mice. Therefore, further investigation of the functions of these metabolites can provide a key component to our understanding of cerebellum’s response to *T. gondii* infection.

## Data Availability Statement

The datasets supporting the findings of this article are included within the paper. The metabolomics data have been deposited in the MetaboLights database (https://www.ebi.ac.uk/metabolights/MTBLS1537).

## Ethics Statement

All mice were handled strictly in accordance with the Animal Ethics Procedures and Guidelines of the People’s Republic of China. The study protocol was reviewed and approved by the Animal Administration and Ethics Committee of Lanzhou Veterinary Research Institute, Chinese Academy of Agricultural Sciences (Permit No. LVRIAEC2017-06).

## Author Contributions

HE, J-JH and X-QZ conceived and designed the study and critically revised the manuscript. JM performed the experiment, analyzed the metabolomics data and drafted the manuscript. J-JH, J-LH and C-XZ helped in data analysis and manuscript revision. All authors contributed to the article and approved the submitted version.

## Conflict of Interest

The authors declare that the research was conducted in the absence of any commercial or financial relationships that could be construed as a potential conflict of interest.

## Acknowledgments

The authors are thankful for the technical assistance provided by BGI-Shenzhen, China.

## Supplementary Material

The Supplementary Material for this article can be found online at: https://www.frontiersin.org/articles/10.3389/fmicb.2020.01555/full#supplementary-material.

## Footnotes

^1^http://www.hmdb.ca/

^2^www.genome.jp/kegg/
