# Supplementary material for: Ultra Performance Liquid Chromatography-Tandem Mass Spectrometry-Based Metabolomics Reveals Metabolic Alterations in the Mouse Cerebellum During Toxoplasma gondii Infection
Source: Front Microbiol. 2020 Jul 10;11:1555. doi: 10.3389/fmicb.2020.01555 (PMC7381283; doi:10.3389/fmicb.2020.01555)
Supplement: Supplementary file 1 [file Data_Sheet_1.docx]

**Table S1 |** Differentially abundant metabolites mapped to the corresponding KEGG metabolic pathways including up- and down-regulated metabolites at 7, 14 and 21 days post infection (dpi) by *Toxoplasma gondii*.

| Metabolite | Log2 fold change | *P* value of *t*-test | VIP | m.z | Retention time (min) | Metabolic pathway |
| --- | --- | --- | --- | --- | --- | --- |
| **Upregulated at 7 dpi** |  |  |  |  |  |  |
| 3,4-Dihydroxyphenylacetic acid | 2.12763328 | 0.0254050 | 3.9046 | 203.01 | 0.76835 | Tyrosine metabolism; Metabolic pathways; Dopaminergic synapse; Cocaine addiction; Amphetamine addiction; Alcoholism |
| Pyroglutamic acid | 1.262373019 | 0.0116520 | 2.4364 | 128.03 | 0.76835 | Glutathione metabolism |
| 7(1)-Hydroxychlorophyll A | 0.756340152 | 0.0122916 | 2.5233 | 909.55 | 10.62433 | Porphyrin and chlorophyll metabolism; Metabolic pathways |
| p-Cresol | 0.422448312 | 0.0245706 | 2.0514 | 91.05 | 10.85935 | Metabolic pathways; Protein digestion and absorption |
| **Downregulated at 7 dpi** |  |  |  |  |  |  |
| Ceramide | -1.77102743 | 0.0429682 | 2.7555 | 598.50 | 11.73210 | Sphingolipid metabolism; Metabolic pathways; Sphingolipid signaling pathway; Leishmaniasis; Neurotrophin signaling pathway; Adipocytokine signaling pathway; AGE-RAGE signaling pathway in diabetic complications; Insulin resistance |
| Nerolidol | -1.116222802 | 0.0364484 | 2.1858 | 212.20 | 5.04582 | Metabolic pathways |
| Lecithin | -0.798617064 | 0.0142575 | 2.7845 | 854.57 | 11.25160 | Glycerophospholipid metabolism; Arachidonic acid metabolism; Linoleic acid metabolism; alpha-Linolenic acid metabolism; Metabolic pathways; Retrograde endocannabinoid signaling; Choline metabolism in cancer |
| Cephalin | -0.625044004 | 0.0484122 | 2.5077 | 842.55 | 11.71268 | Glycosylphosphatidylinositol(GPI)-anchor biosynthesis; Glycerophospholipid metabolism; Metabolic pathways; Regulation of autophagy; Retrograde endocannabinoid signaling |
| Oleic acid | -0.530739153 | 0.0187642 | 2.2860 | 300.29 | 6.92742 | Fatty acid biosynthesis; Biosynthesis of unsaturated fatty acids |
| 2-Lysophosphatidylcholine | -0.428565884 | 0.0106977 | 2.1996 | 550.33 | 11.19438 | Glycerophospholipid metabolism; Choline metabolism in cancer |
| **Upregulated at 14 dpi** |  |  |  |  |  |  |
| Galactosylsphingosine | 1.612069358 | 0.0001796 | 3.9403 | 460.33 | 7.76892 | Sphingolipid metabolism |
| Linoleic acid | 1.369215194 | 0.0244308 | 2.3431 | 279.23 | 8.75972 | Linoleic acid metabolism; Biosynthesis of unsaturated fatty acids; Metabolic pathways |
| Phosphatidylserine | 1.018634174 | 0.0381789 | 3.3596 | 836.54 | 9.62477 | Glycine, serine and threonine metabolism; Glycerophospholipid metabolism; Metabolic pathways; Leishmaniasis; Amoebiasis; Systemic lupus erythematosus |
| Docosahexaenoic acid | 0.774080691 | 0.0204978 | 2.4109 | 327.23 | 8.00393 | Biosynthesis of unsaturated fatty acids |
| Diacylglycerol | 0.704783453 | 0.0267321 | 2.1561 | 631.51 | 10.34913 | EGFR tyrosine kinase inhibitor resistance; MAPK signaling pathway; ErbB signaling pathway; Ras signaling pathway; Rap1 signaling pathway; Calcium signaling pathway; cAMP signaling pathway; Chemokine signaling pathway; NF-kappa B signaling pathway; HIF-1 signaling pathway; Sphingolipid signaling pathway; Phospholipase D signaling pathway; Adrenergic signaling in cardiomyocytes; Vascular smooth muscle contraction; VEGF signaling pathway; Gap junction; Platelet activation; Natural killer cell mediated cytotoxicity; T cell receptor signaling pathway; B cell receptor signaling pathway; Fc epsilon RI signaling pathway; Fc gamma R-mediated phagocytosis; Circadian entrainment; Long-term potentiation; Neurotrophin signaling pathway; Retrograde endocannabinoid signaling; Glutamatergic synapse; Cholinergic synapse; Serotonergic synapse; Dopaminergic synapse; Long-term depression; Inflammatory mediator regulation of TRP channels; Insulin secretion; GnRH signaling pathway; Estrogen signaling pathway; Melanogenesis; Thyroid hormone synthesis; Thyroid hormone signaling pathway; Adipocytokine signaling pathway; Oxytocin signaling pathway; Regulation of lipolysis in adipocytes; Aldosterone synthesis and secretion; Insulin resistance; AGE-RAGE signaling pathway in diabetic complications; Endocrine and other factor-regulated calcium reabsorption; Salivary secretion; Gastric acid secretion; Pancreatic secretion; Carbohydrate digestion and absorption; Fat digestion and absorption; African trypanosomiasis; Amoebiasis; Pathways in cancer; Glioma; Non-small cell lung cancer; Choline metabolism in cancer |
| Calcidiol | 0.691355492 | 0.01761 | 2.3279 | 401.34 | 9.93952 | Steroid biosynthesis; Metabolic pathways; Tuberculosis |
| Sphingosyl-phosphocholine | 0.687150294 | 0.0113716 | 2.5192 | 448.34 | 7.97535 | Sphingolipid metabolism |
| 27-Deoxy-5b-cyprinol | 0.670024511 | 0.0242327 | 2.0877 | 454.39 | 8.52468 | Primary bile acid biosynthesis; Metabolic pathways |
| Arachidonic acid | 0.660563586 | 0.0114548 | 2.2795 | 303.23 | 8.00393 | Arachidonic acid metabolism; Linoleic acid metabolism; Biosynthesis of unsaturated fatty acids; Metabolic pathways; Vascular smooth muscle contraction; Platelet activation; Fc epsilon RI signaling pathway; Fc gamma R-mediated phagocytosis; Retrograde endocannabinoid signaling; Serotonergic synapse; Long-term depression; Inflammatory mediator regulation of TRP channels; GnRH signaling pathway; Ovarian steroidogenesis; Oxytocin signaling pathway; Regulation of lipolysis in adipocytes; Aldosterone synthesis and secretion; Leishmaniasis; Amoebiasis |
| L-Palmitoylcarnitine | 0.644686943 | 0.0124247 | 2.3402 | 400.34 | 8.08965 | Fatty acid degradation; Fatty acid metabolism |
| Lecithin | 0.623679608 | 0.0230554 | 2.2131 | 760.58 | 9.6101 | Glycerophospholipid metabolism; Arachidonic acid metabolism; Linoleic acid metabolism; alpha-Linolenic acid metabolism; Metabolic pathways; Retrograde endocannabinoid signaling; Choline metabolism in cancer |
| 2-Lysophosphatidylcholine | 0.501617481 | 0.0066129 | 1.7824 | 520.34 | 8.7597 | Glycerophospholipid metabolism; Choline metabolism in cancer |
| Chitin | 0.48326116 | 0.0034202 | 1.6154 | 718.28 | 8.8883 | Amino sugar and nucleotide sugar metabolism; Metabolic pathways |
| 4,4-Dimethyl-5a-cholesta-8-en-3b-ol | 0.406971971 | 0.0362624 | 1.1947 | 415.39 | 9.09555 | Steroid biosynthesis |
| 17alpha,21-Dihydroxypregnenolone | 0.39791246 | 0.0331238 | 1.5908 | 347.22 | 9.65050 | Steroid hormone biosynthesis |
| Sulfatide | 0.358958826 | 0.0483117 | 1.6373 | 762.52 | 10.52432 | Sphingolipid metabolism; Metabolic pathways |
| Norfloxacin | 0.333881659 | 0.0090873 | 2.0373 | 337.17 | 9.6536 | ABC transporters |
| **Downregulated at 14 dpi** |  |  |  |  |  |  |
| Uridine | -1.328434874 | 0.0338306 | 3.6166 | 243.06 | 0.76835 | Pyrimidine metabolism; Metabolic pathways |
| 5alpha-Cholesta-7,24-dien-3beta-ol | -0.594225422 | 0.0219616 | 1.3968 | 385.35 | 9.6536 | Steroid biosynthesis; Metabolic pathways |
| Tabersonine | -0.478547174 | 0.0398621 | 1.693 | 371.15 | 9.48692 | Metabolic pathways |
| Sphingomyelin | -0.425268737 | 0.0410131 | 1.3687 | 729.59 | 11.41527 | Sphingolipid metabolism; Metabolic pathways; Sphingolipid signaling pathway |
| Ethylbenzene | -0.344098678 | 0.0283033 | 1.626 | 129.07 | 9.85368 | Metabolic pathways |
| **Upregulated at 21 dpi** | | | | | | |
| 27-Deoxy-5b-cyprinol | 1.042574302 | 0.001179 | 3.029 | 454.39 | 8.52468 | Primary bile acid biosynthesis; Metabolic pathways |
| Phosphatidylethanolamine | 0.982655956 | 0.0315617 | 2.6191 | 707.57 | 10.90222 | Retrograde endocannabinoid signaling; Glycerophospholipid metabolism; Regulation of autophagy; Glycosylphosphatidylinositol(GPI)-anchor biosynthesis; Metabolic pathways |
| Allotetrahydrodeoxycorticosterone | 0.929033479 | 0.0055692 | 3.4891 | 357.24 | 9.02365 | Steroid hormone biosynthesis |
| Calcitetrol | 0.872631791 | 0.0142403 | 2.7337 | 450.36 | 8.13252 | Steroid biosynthesis; Metabolic pathways |
| Cytidine | 0.814837498 | 0.0242102 | 2.0896 | 242.08 | 0.61903 | Pyrimidine metabolism; Metabolic pathways |
| Eucalyptol | 0.715454127 | 0.0432313 | 2.301 | 137.13 | 10.4386 | Inflammatory mediator regulation of TRP channels |
| Docosahexaenoic acid | 0.703898038 | 0.0291186 | 1.4977 | 327.23 | 8.00393 | Biosynthesis of unsaturated fatty acids |
| 13-OxoODE | 0.638768944 | 0.0070058 | 2.0555 | 317.21 | 8.48182 | Linoleic acid metabolism |
| 5-Hydroxyconiferaldehyde | 0.56627937 | 0.0322459 | 2.5185 | 195.06 | 5.73017 | Metabolic pathways |
| Arachidonate | 0.565694652 | 0.0209242 | 1.4625 | 303.23 | 8.00393 | Arachidonic acid metabolism; Linoleic acid metabolism; Biosynthesis of unsaturated fatty acids; Metabolic pathways; Vascular smooth muscle contraction; Platelet activation; Fc epsilon RI signaling pathway; Fc gamma R-mediated phagocytosis; Leishmaniasis; Amoebiasis; Aldosterone synthesis and secretion; Retrograde endocannabinoid signaling; Serotonergic synapse; Long-term depression; Inflammatory mediator regulation of TRP channels; GnRH signaling pathway; Ovarian steroidogenesis; Oxytocin signaling pathway; Regulation of lipolysis in adipocytes |
| L-Palmitoylcarnitine | 0.51470301 | 0.0211203 | 1.7939 | 400.34 | 8.08965 | Fatty acid degradation; Fatty acid metabolism |
| Androstan-3alpha,17beta-diol | 0.447420505 | 0.0034798 | 2.2404 | 293.25 | 8.92362 | Steroid hormone biosynthesis |
| S-Lactoylglutathione | 0.446362112 | 0.0434388 | 1.7637 | 397.14 | 8.49612 | Pyruvate metabolism |
| Phenethyl alcohol | 0.390997784 | 0.0376825 | 1.6072 | 105.07 | 10.4386 | Phenylalanine metabolism |
| Sphingosyl-phosphocholine | 0.385707125 | 0.0481967 | 1.3457 | 448.34 | 7.97535 | Sphingolipid metabolism |
| 9-OxoODE | 0.313245852 | 0.0034609 | 1.7625 | 277.22 | 8.48182 | Linoleic acid metabolism |
| 2-Chloro-3-oxoadipate | 0.270708334 | 0.0100007 | 1.3141 | 192.99 | 6.5781 | Metabolic pathways |
| Traumatic acid | 0.268673925 | 0.0429967 | 1.3195 | 227.13 | 7.09885 | alpha-Linolenic acid metabolism |
| Chenodeoxycholic acid | 0.264716569 | 0.0405249 | 1.1786 | 391.29 | 9.92195 | Primary bile acid biosynthesis; Metabolic pathways; Bile secretion |
| **Downregulated at 21 dpi** |  |  |  |  |  |  |
| 5,6-Epoxytetraene | -1.315809634 | 0.0393818 | 3.2272 | 357.2 | 7.88677 | Arachidonic acid metabolism |
| Phosphatidylserine | -1.224317298 | 0.045255 | 3.5048 | 842.58 | 10.67363 | Glycine, serine and threonine metabolism; Glycerophospholipid metabolism; Metabolic pathways; Leishmaniasis; Amoebiasis; Systemic lupus erythematosus |
| Calcidiol | -1.114035243 | 0.0424188 | 3.8157 | 401.34 | 9.93952 | Steroid hormone biosynthesis; Metabolic pathways |
| Cortolone | -0.963250524 | 0.0429697 | 2.6489 | 389.23 | 7.7832 | Steroid hormone biosynthesis |
| Lecithin | -0.818324939 | 0.0471112 | 2.8721 | 870.53 | 9.6101 | Glycerophospholipid metabolism; Arachidonic acid metabolism; Linoleic acid metabolism; alpha-Linolenic acid metabolism; Metabolic pathways; Retrograde endocannabinoid signaling; Choline metabolism in cancer |
| Linoleic acid | -0.759014096 | 0.0362182 | 1.8439 | 279.23 | 8.8883 | Linoleic acid metabolism; Biosynthesis of unsaturated fatty acids; Metabolic pathways |
| Pravastatin | -0.692921261 | 0.0201393 | 2.3018 | 407.24 | 7.11313 | Bile secretion |
| 5,6-Epoxy-8,11,14-eicosatrienoic acid | -0.668180152 | 0.0064098 | 1.6234 | 319.23 | 9.15188 | Arachidonic acid metabolism; Metabolic pathways; Vascular smooth muscle contraction; Serotonergic synapse; Inflammatory mediator regulation of TRP channels; Ovarian steroidogenesis |
| Fexofenadine | -0.59596886 | 0.0238887 | 2.1803 | 524.28 | 7.91822 | Bile secretion |
| Rhodovibrin | -0.557879241 | 0.00365 | 1.2191 | 619.42 | 9.97125 | Metabolic pathways |
| Palmitoleic acid | -0.50758276 | 0.0029166 | 1.7592 | 272.26 | 8.73113 | Fatty acid biosynthesis |
| Estriol | -0.449493299 | 0.0236978 | 1.9759 | 289.18 | 7.96107 | Steroid hormone biosynthesis; Metabolic pathways |
| 2-Lysophosphatidylcholine | -0.430703354 | 0.0088237 | 1.7665 | 568.34 | 8.88828 | Glycerophospholipid metabolism; Choline metabolism in cancer |
| Phosphatidic acid | -0.402588012 | 0.0296747 | 1.776 | 452.28 | 8.70257 | Glycerolipid metabolism; Glycerophospholipid metabolism; Metabolic pathways; Choline metabolism in cancer; cAMP signaling pathway; Phosphatidylinositol signaling system; Phospholipase D signaling pathway; Fc gamma R-mediated phagocytosis; GnRH signaling pathway; Fat digestion and absorption; Pathways in cancer; Pancreatic cancer |
| Xanthoxin | -0.363827833 | 0.038251 | 1.765 | 233.15 | 7.18457 | Metabolic pathways |
| Sphingosine | -0.358638944 | 0.0189302 | 1.6477 | 300.29 | 9.28815 | Sphingolipid metabolism; Metabolic pathways; Sphingolipid signaling pathway; Apoptosis |
| 2-Arachidonylglycerol | -0.336609835 | 0.0283267 | 1.3476 | 361.27 | 8.80257 | Neuroactive ligand-receptor interaction; Retrograde endocannabinoid signaling |
| Capric acid | -0.320846479 | 0.0151594 | 1.7822 | 195.14 | 7.19885 | Fatty acid biosynthesis; Metabolic pathways |
| 2'-N-Acetylparomamine | -0.315271013 | 0.0198314 | 1.0173 | 400.15 | 9.95697 | Butirosin and neomycin biosynthesis |
| Vitamin A | -0.311864412 | 0.0330273 | 1.6314 | 287.24 | 9.21007 | Retinol metabolism; Metabolic pathways; Vitamin digestion and absorption |
| 1,2-Dehydroreticuline | -0.299737855 | 0.0475177 | 1.392 | 311.15 | 9.45962 | Metabolic pathways |
| Tryptophol | -0.297431104 | 0.0081563 | 1.4063 | 184.07 | 9.19572 | Tryptophan metabolism |
| (-)-alpha-Terpineol | -0.290532899 | 0.026408 | 1.6639 | 137.13 | 9.62477 | Metabolic pathways |
